# Is calcium a link between inflammatory bone resorption and heart disease?

**DOI:** 10.7554/eLife.83841

**Published:** 2022-12-29

**Authors:** Gordon L Klein

**Affiliations:** 1 Department of Orthopaedic Surgery and Rehabilitation, The University of Texas Medical Branch at Galveston Galveston United States; Icahn School of Medicine at Mount Sinai United States; Icahn School of Medicine at Mount Sinai United States

**Keywords:** cardiovascular disease, chronic inflammation, calcium-sensing receptor, burns, calcium

## Abstract

Several epidemiologic studies associate bone-resorbing chronic inflammatory conditions with increased risk of atherosclerotic heart disease. These include post-menopausal osteoporosis, spinal cord injury, rheumatoid arthritis, and osteoarthritis. Additional studies have noted that the use of anti-resorptive agents following hip fracture, during rheumatoid arthritis, and prior to intensive care management have resulted in reduced overall mortality and mortality from cardiovascular disorders. The careful study of burn patients has allowed us to detect that children and adolescents have a mechanism that protects them from the entry of calcium into the circulation following inflammatory bone resorption. That is, they respond to pro-inflammatory cytokines by up-regulating the parathyroid calcium-sensing receptor (CaSR) with consequent development of hypocalcemic hypoparathyroidism and hypercalciuria. As extracellular calcium appears to exacerbate and/or prolong the inflammatory response, this responsiveness of the CaSR to inflammatory cytokines may be the factor that reduces cardiovascular morbidity and mortality. In adults with chronic inflammatory conditions, the ability of the CaSR to respond to pro-inflammatory cytokines is lost, suggesting that the calcium that enters the circulation following inflammatory bone resorption may persist in the circulation, entering the small coronary blood vessels and favoring the formation of coronary artery calcification, inflammation, and consequent cardiovascular disease.

## Introduction

Epidemiologic studies can call attention to relationships among health conditions that are not intuitively apparent and for which no clear causative mechanism can provide an explanation. Yet the confluence of evidence points toward the existence of a relationship that must be further explored. Such is the case with inflammatory bone resorption and cardiovascular disease. To examine this relationship, I will first discuss the epidemiologic studies that relate chronic inflammatory conditions, such as post-menopausal osteoporosis, spinal cord injury, rheumatoid arthritis, and osteoarthritis to cardiovascular disease. Following this, I will look at the smaller amount of evidence linking anti-resorptive agents, such as nitrogen-containing bisphosphonates, to reduced cardiovascular morbidity and mortality. Finally, I will examine evidence from burn injury, a condition which itself manifests a robust systemic inflammatory response, which suggests a possible mechanism that may link chronic inflammatory conditions with cardiovascular disease.

### The relationship between chronic inflammatory conditions and cardiovascular disease: evidence from epidemiologic studies

#### Post-menopausal osteoporosis

Post-menopausal osteoporosis is normally considered a condition caused by a lack of estrogen leading to an increase in bone resorption and a decrease in bone formation, resulting in net bone loss and an increase in fragility fractures, those occurring following a fall from a standing height. However, as noted by Weitzmann and Pacifici, 2006, estrogen can suppress osteoclastogenesis and osteoclastogenesis sensitivity to the receptor activator of the ligand of NFκB (RANKL). Estrogen receptors can bind to transcription factors that suppress cytokine production, such as interleukin (IL)-6 and tumor necrosis factor (TNF)-α. Thus, estrogen deficiency is associated with de-repression of inflammatory cytokines and increased inflammatory bone loss. In the Rotterdam Study of the relationship between bone health and coronary artery calcification Campos-Obando et al., 2015 studied changes over time in bone density and coronary artery calcification in 582 men and 689 women. While there was no correlation in men, each 1% increase in bone loss was associated with an increase in coronary artery calcification in women with low estradiol levels, suggesting that low estradiol has a role in this relationship. Failure of hormone replacement to reduce mortality in post-menopausal women compared to bisphosphonate treatment, as reported by Center et al., 2011 may relate to the inability of the doses of hormone replacement to suppress the production of inflammatory cytokines. However, this must remain speculative.

Lee et al., 2016a reported on the association of osteoporosis and cardiovascular disease in 863 post-menopausal women, and found a significant correlation between low bone mineral density and high coronary artery calcium score (p = 0.015) and high obstructive coronary artery disease (p = 0.002). Additionally, Slinin et al., 2011 in the Multiple Outcomes of Raloxifene Evaluation, the MORE study evaluated 7259 post-menopausal women and found an independent association between higher baseline serum calcium levels and a higher rate of cardiovascular events, with no association between baseline serum phosphorus and cardiovascular events.

#### Spinal cord injury

Spinal cord injury is another condition that produces inflammation. Hellenbrand et al., 2021 have reviewed the timeline of the inflammatory response in the rodent model. Its onset is acute, with cytokine up-regulation occurring within hours of the original injury, and involving IL-1, IL-6, and TNF-α. These cytokines are produced by microglia, astrocytes, and peripherally derived macrophages. The resulting inflammation is both local and systemic. Qin et al., 2010 describe the resorptive bone loss that occurs acutely at and below the level of spinal cord injury while bone above the lesion is intact. The systemic inflammation and resorptive bone loss occur over the same period. More recently, Cirnigliaro et al., 2017 identified bone loss at the proximal tibia and distal femur in spinal cord injury. With regard to cardiovascular disease, Bauman and Spungen, 2008 note a higher prevalence and greater degree of coronary arterial calcification occurs in patients with spinal cord injury when matched to patients without spinal cord injury by age, gender, ethnicity, and conventional risk factors. More recently, Peterson et al., 2021 examined the 5-year incidence of all cardiac and metabolic abnormalities with spinal cord injury compared to the general population without spinal cord injury from national insurance claims. The database included 9000 patients with spinal cord injury compared to 1.5 million without. Subjects in the control group were screened for non-spinal cord injury-related neurologic disabilities and those identified were eliminated. Patients in the control group with cardiovascular comorbidities at the outset of follow-up period were also excluded from the analysis. Adults with traumatic spinal cord injury had a greater 5-year incidence of cardiac and metabolic abnormalities, 56% compared to 34% with an adjusted hazard ratio for heart failure in spinal cord injury population of 3.55, exceeding any purely metabolic conditions.

#### Rheumatoid arthritis

In rheumatoid arthritis, Karpouzas et al., 2020 reported that over a 7-year period, coronary artery calcium score and plaque progression increased in patients of older age and with higher cumulative inflammation than patients who demonstrated no progression. Liao, 2017 in a review of rheumatoid arthritis and cardiovascular disease noted that the incidence of cardiovascular disease in patients with rheumatoid arthritis was 1.5–2 times higher than in age- and sex-matched individuals in the general population. Liao also notes that the IL-6 receptor may be a common factor in both chronic inflammation and cardiovascular disease pathogenesis, as reported by Hingorani and Casas (Swerdlow et al., 2012). This latter study used single-nucleotide polymorphisms of the IL-6 receptor gene to evaluate likelihood and efficacy and safety of IL-6 receptor inhibition to prevent cardiovascular disease. The Interleukin-6 Receptor Mendelian Randomization Consortium compared the genetic effects of the SNPs with the effects of the drug tocilizumab, which is used in IL-6 receptor blockade in patients with rheumatoid arthritis. An IL-6 SNP was identified that appeared consistent with the IL-6 receptor blockade effects of tocilizumab, suggesting that the IL-6 receptor was involved in the pathogenesis of cardiovascular disease. These features were confirmed in a review by England et al., 2018 of 2687 reports in the literature.

#### Osteoarthritis

In 2016, Wang et al., 2016 published a meta-analysis of 15 articles covering 358,944 subjects, including 80,911 with osteoarthritis and 29,213 patients with cardiovascular disease. The overall risk of cardiovascular disease in osteoarthritis was calculated to be 24% greater than in the general population. Also in 2016, a systematic review and meta-analysis by Hall et al., 2016 reviewed studies including over 32 million patients. Those with osteoarthritis were three times as likely to have either cardiac failure or ischemic heart disease than matched cohorts without osteoarthritis. In a more current meta-analysis by Macêdo et al., 2022, the authors reviewed 49 studies including 500,000 patients and 688,000 controls. Hip and knee osteoarthritis increased the odds ratio for both sub-clinical atherosclerosis, 1.15, and cardiovascular disease, 1.13, but not cardiovascular mortality. Interestingly, in a recent two-way Mendelian analysis of causation between osteoarthritis and coronary artery disease and myocardial infarction, Xu et al., 2022 found no evidence supporting causation of one by the other.

#### Burns

While I did not originally include burns as a condition that includes chronic inflammation, our observations in burns, as you will see, provide the evidence for a link between chronic inflammatory conditions and cardiovascular disease. The actual duration of inflammation following burn injury has not been documented, but evidence of elevated cytokines in the circulation exists for as long as 3 years following a severe burn injury (Finnerty et al., 2013). In support of burns as possibly providing a mechanism linking chronic inflammatory disease and cardiovascular disease, a study published by Duke et al., 2017, reviewed age- and gender-matched groups of patients with burn injury, 30,000, non-burn injury, 28,000, and uninjured patients, 123,000, for incidence and duration of cardiovascular-related hospital admissions over 5 years. The authors found that burn patients had a higher rate of initial admissions for cardiovascular conditions than non-burn-injured patients and that both groups had a higher rate of cardiovascular admissions than did uninjured patients. To date, we do not know the mechanism for this increased incidence of hospitalization for burn patients manifesting cardiovascular disease.

### Epidemiologic studies of bisphosphonates and mortality

A relatively recent group of reports on epidemiologic studies of the effects of the use of bisphosphonates in chronic disease and critical illness identified an increase in longevity with bisphosphonate use. To date, it is unclear what these findings mean for patients. However, after I review these studies, I will attempt to combine them with the discussions in the previous sections.

#### Bisphosphonates and reduced mortality

In 2007, Lyles et al., 2007 published the results of a trial of zolendronate versus placebo in 2127 patients recruited from the HORIZON Recurrent Fracture Trial. They found a 28% reduction in all-cause mortality. The significance of the finding was unclear. Of potentially greater importance, however, is a larger study reported in 2013 by Wolfe et al., 2013 of more than 19,000 patients in the National Data Bank for Rheumatic Disease. The authors examined the risk of myocardial infarction longitudinally from 2003 to 2011 and the adjusted risk for myocardial infarction in bisphosphonate users compared to non-users was 0.72. In support of these findings, a study by Lee et al., 2016b examined retrospectively the cases of 7830 critically ill patients in Sydney, Australia from 2003 to 2014. These patients had a variety of critical illnesses involving cardiovascular, neurological, gastrointestinal, toxic metabolic, as well as traumatic conditions. The main outcome was in-hospital mortality. Patients who received pre-admission bisphosphonates were older and had a greater number of comorbidities. Nevertheless, these patients had a lower in-hospital morbidity than non-users of bisphosphonates. Bisphosphonate users had a mortality rate ratio of 0.41 after adjusting for age, sex, principal diagnosis, comorbidities, admitting unit, and admission year. Bone density reduction was −3 ± 13% per week among bisphosphonate users compared to −15 ± 14% per week among non-users (p < 0.01).

An earlier study by Center et al., 2011 examined the effect of bisphosphonates versus hormone therapy versus calcium and vitamin D prospectively between 1989 and 2007 in 1223 women and 819 men over age 60 in the Dubbo Osteoporosis Epidemiology Study. Of the 325 women on treatment, 106 were receiving bisphosphonates, 77 hormone replacement therapy, and 142 calcium and vitamin D. Only 37 men were on treatment, 15 on bisphosphonates and 22 on calcium and vitamin D. Mortality rate was lower for women receiving bisphosphonates, 0.3 hazard ratio, but not for those receiving hormone replacement or calcium and vitamin D. Mortality rate was independent of fractures, but in the fracture group mortality was still lower in the bisphosphonate-treated group.

#### Summary

From these studies, bisphosphonate use appears to reduce mortality risk in patients with post-menopausal osteoporosis, and in rheumatoid arthritis, especially cardiovascular disease-related, as well as in critical illness in an intensive care setting. Note that other therapies are not specifically directed to bone resorption, so that it is possible that bone resorption is somehow involved in cardiovascular mortality. I have also cited evidence in the sections ‘Post-menopausal osteoporosis, Spinal cord injury, Rheumatoid arthritis, Osteoarthritis, and Burns’ that inflammation raises the risk of cardiovascular disease. It is now time to describe the studies in burns that suggest a mechanism linking inflammation to bone resorption to increased cardiovascular risk.

#### Burns as a model of acute and sustained inflammation

I have already shown from the study of Duke et al., 2017 that burn injury results in later hospital admissions for cardiovascular disease. Burns are also a source of acute inflammation beginning on day 1 and resulting from the destruction of the skin barrier to microorganism entry into the body. In pediatric burn injury, circulating IL-1β increases threefold and IL-6 increases one hundredfold by 2 weeks post-burn (Klein et al., 1995). In a sheep model of burn injury, backscatter, scanning electron microscopy demonstrates the appearance of scalloping at the iliac crest bone surface by day 5 post-burn. Scalloping of the bone surface is a histologic feature of bone resorption. Also, the urinary C-telopeptide of type I collagen, CTx, a biomarker of bone resorption, is elevated by day 1 post-burn (Klein et al., 2014), demonstrating that inflammation and resorptive bone loss both occur acutely and over the same time period.

#### Calcium metabolism and the parathyroid calcium-sensing receptor

In vitro studies by Nielsen et al., 1997, Toribio et al., 2003 and later by Canaff et al., 2008 demonstrated that pro-inflammatory cytokines IL-1 and IL-6 were capable of up-regulating the parathyroid and renal calcium-sensing receptors (CaSRs) in vitro. I will focus attention on the parathyroid CaSR for purposes of this review. This transmembrane G-protein-coupled receptor on the parathyroid chief cell, when up-regulated, lowers the amount of circulating calcium necessary to suppress PTH secretion by the parathyroid gland, thus creating a hypocalcemic hypoparathyroid state, with increased urinary calcium excretion. We have previously shown that in a sheep model of burn injury, there is a 50% up-regulation of the parathyroid CaSR within 48 hr of the burn (Murphey et al., 2000). The associated hypocalcemic hypoparathyroidism was documented as well in the pediatric burn population (Klein et al., 1997). The apparent coincidence of post-burn bone resorption and up-regulation of the CaSR in the sheep model suggested that the hypocalcemic hypoparathyroidism was protective against the influx of calcium from resorbing bone into the circulation, allowing the excretion of excess calcium.

However, the full protective effect of the CaSR up-regulation became apparent when our in vitro studies of peripheral blood mononuclear cells in culture with varying amounts of medium calcium revealed significant positive and inverse correlations of medium calcium with various chemokines produced by the peripheral blood mononuclear cells (Klein et al., 2016). These findings suggested that extracellular calcium was a potent regulator of immune cell chemokine production. Our results were reinforced by the report of Rossol et al., 2012 showing that extracellular calcium can stimulate the NLRP3 inflammasome to cause monocytes and macrophages of the innate immune system to produce IL-1. Evidence reviewed by Abbate et al., 2020 supports a role of the NLRP3 inflammasome and IL-1 in the pathogenesis of atherosclerosis.

Thus, CaSR up-regulation by pro-inflammatory cytokines would appear to have a protective effect on the cardiovascular system of children following burn injury. However, the responsiveness of the parathyroid CaSR to pro-inflammatory cytokines in adult burn injury appears to be lost. Table 1 shows that in contrast to burned children, adults suffering burn injury do not manifest the hypocalcemic hypoparathyroidism characteristic of pediatric patients. Adults with severe burns were normocalcemic or mildly hypercalcemic and either euparathyroid or mildly hyperparathyroid. It is important to note in this regard that only values for ionized calcium are used since following burn there is an acute fall in circulating albumin and recovery of normal plasma constitutive proteins does not recur for at least 6 months post-burn. Thus, low total serum calcium could be due to low circulating concentrations of binding proteins rather than to abnormalities in calcium metabolism per se. Blood ionized calcium levels sort this out (Krajewski et al., 2020). The data in this latter study are consistent with the proposed model of burn-associated hypocalcemia in children. These hypocalcemic values would result from up-regulation of the parathyroid CaSR by pro-inflammatory cytokines. In burned adults, ionized calcium is normal to slightly elevated, which indicates non-responsiveness of the parathyroid CaSR to the pro-inflammatory cytokines.

**Table 1.** Blood ionized calcium and parathyroid hormone concentration in burns patients.

Source	Pediatric/adult	Mean (range) ionized Ca (normal)	Mean (range) PTH (normal)
Lovén et al., 1984	Adult	(1.03–1.12) (1.1–1.3) mmol/l	1.4–2.3 (1.1–2.5) µmol/l
Coté et al., 1988	Pediatric	(1.06–1.20) mmol/l*	---------------
Klein et al., 1993	Adult	1.15 ± 0.06 SD (1.00–1.15) mmol/l	114 ± 96 (9–66) pg/ml
Klein et al., 1997	Pediatric	1.08 ± 0.03 (1.12–1.37) mmol/l	7 ± 3 (15–55) pg/ml
Dolecek et al., 2003	Adult	0.96–1.18 mmol/l*	17–42 pg/ml*
Gottschlich et al., 2017	Pediatric	------------	12.5 ± 7(15–65) pg/ml
Rousseau et al., 2015	Adult	0.98–1.26 (1.14–1.3) mmol/l	10–114 (4–26) pg/ml
Muschitz et al., 2016	Adult	1.11^†^ (1.15–1.35) mmol/l	50.1^†^ (15–65) pg/ml
Sobouti et al., 2016	Pediatric	1.29 ± 0.06* mmol/l	11.1 ± 5.8 pg/ml M* 10.9 ± 7.6 pg/ml F*
Mohammadi and Shafaeipour, 2021	Adult	---------------	50 ± 26 (15–65) pg/ml

Key: ‐‐‐‐‐‐ = value not given.

SD = all values with + = mean and standard deviation.

* Normal range not given.

† Median value.

#### Possible consequences of the age-related loss of parathyroid CaSR responsiveness

It is not known why the CaSR responsiveness to pro-inflammatory cytokines is lost or precisely when this happens. We know that even in small pediatric burns, that is from 1% to 20%, children and adolescents up through age 19 continue to exhibit the hypocalcemic response to burn injury (Klein, 2022). Therefore, the earliest age range in which the CaSR responsiveness can be lost is in the 20s. This age range roughly corresponds to the period of acquisition of peak bone mass. While any relationship between the two events is currently speculative, any change in calcium handling by the body at this age has yet to be investigated but might prove revealing.

Without knowing the cause of this change, the consequences can perhaps involve other chronic inflammatory conditions as previously discussed in the section ‘The relationship between chronic inflammatory conditions and cardiovascular disease: evidence from epidemiologic studies’. Thus, in addition to burns, spinal cord injury, post-menopausal osteoporosis, rheumatoid arthritis, and osteoarthritis all involve inflammatory bone resorption with entry of resorbed calcium into the circulation but perhaps without the ability to excrete the excess calcium and lower circulating calcium concentration. This excess calcium then can enter small vessels, such as the coronaries, and, as suggested by Abbate et al., 2020, increase atherosclerotic plaque formation and small vessel calcification. The excess calcium might even interact with the endothelial CaSR (Klein et al., 2008; Hannan et al., 2018) in these small vessels causing increased vascular tone. The epidemiologic evidence mentioned earlier would support the increased coronary artery calcium accumulation and consequent atherosclerotic disease in these populations.

Should intravascular calcium retention be a possible etiology it might be worthwhile examining two possible avenues of prevention. The first would be referral of patients with any of the above conditions for cardiology evaluation regarding evidence of existing cardiovascular disease. The second could be investigation of the use of a calcimimetic to see if it can re-activate the adult parathyroid CaSR. If that were not successful, perhaps the earlier use of anti-resorptives could help lower the risk of atherogenesis.

#### Unanswered questions: the role of hypercalcemia in coronary artery disease and vascular calcification

While the above-mentioned clinical conditions appear to be associated with calcium retention and an increased incidence of cardiovascular disease, the argument for hypercalcemia as a cause of cardiovascular disease is not as clear. We have already mentioned that post-menopausal osteoporosis has been shown to exhibit correlations between higher serum calcium concentrations and a higher incidence of cardiovascular disease in a report by Slinin et al., 2011. However, Bilezikian, 2018 points out that primary hyperparathyroidism is the primary cause of hypercalcemia in post-menopausal osteoporosis, raising the question as to whether primary hyperparathyroidism or inflammation, as previously mentioned (Weitzmann and Pacifici, 2006) and more recently summarized in a review by Wu et al., 2021 is responsible for the cardiovascular calcifications seen in this condition. In addition, hemodialysis patients with renal osteodystrophy demonstrate both high serum calcium concentration from secondary hyperparathyroidism and a robust inflammatory response and arterial calcifications (Chertow et al., 2012), although the pathogenesis is likely multifactorial.

With regard to rheumatoid arthritis and osteoarthritis, the evidence is more limited. In a cross-sectional study of ionized calcium in 146 patients with rheumatoid arthritis, Oelzner et al., 2006 found ionized hypercalcemia in 30%, leaving 70% with normocalcemia. They found that markers of inflammation were significantly higher in those with ionized hypercalcemia and bone density was lower. The findings are consistent with greater inflammation-mediated bone loss. This is the only study I have found that investigates ionized hypercalcemia. However, normocalcemia may be inappropriately high if patients have lower serum concentrations of albumin in these inflammatory conditions. I have not come across a longitudinal study of patterns of calcium concentration, ionized or albumin-corrected, in any of these inflammatory conditions. In spinal cord injury, hypercalcemia is present but is documented to be associated with immobilization (Massagli and Cardenas, 1999). Again, there is an open question as to whether both immobilization and inflammation contribute to the vascular calcification seen in this setting.

Thus, while that evidence which exists would support hypercalcemia’s association with inflammatory disease, it is presently unclear whether hypercalcemia is a necessary antecedent to cardiovascular disease. One clue can be gained by examining function of the vascular smooth muscle CaSR, which is reported to be protective from myocardial infarctions in the review by Hannan et al., 2018. When the vascular smooth muscle CaSR is underexpressed, such as in hypercalcemia, protection against myocardial infarction should be reduced. Also, given the failure of the parathyroid CaSR to respond to inflammatory cytokines in adults, what is the nature of the equilibrium of circulating calcium with adjacent tissues? The appearance of vascular calcifications would need to be studied longitudinally and correlated with either blood ionized calcium or albumin-corrected calcium in the aforementioned patient populations in order to better understand the dynamics of tissue/vascular calcification in relation to blood concentration of calcium.
